# Cannabis in road traffic – a retrospective analysis to identify possible cut-off-values

**DOI:** 10.1007/s12024-025-01133-1

**Published:** 2025-11-17

**Authors:** A. Ludwig, U. Küpper, M. Lau, A. Holzer, T. Wulff, B. Hartung

**Affiliations:** 1 Institute of Legal Medicine, University Hospital Essen, Essen, Germany; 2 Mathematical Institute, Heinrich Heine University Düsseldorf, Düsseldorf, Germany

**Keywords:** Driving under influence, Cannabis, THC threshold, Cannabis legalization, Alcohol

## Abstract

In light of the recent partial legalization of cannabis in Germany and the associated adjustment of the legal threshold for THC serum concentration from 1 ng/ml to 3.5 ng/ml, we investigated whether a specific THC serum concentration or cannabis influence factor (CIF) value could be identified, above which behavioural and driving impairments significantly increase. We retrospectively analysed cases from 2020 to 2021, in which blood samples had been submitted for toxicological analysis from drivers suspected of operating a vehicle under the influence of cannabis alone (DUIC) or in combination with alcohol (DUIAC). 740 cases were evaluated with respect to behavioural and driving impairments, serum concentration of THC and its metabolites, CIF values, and—in cases involving co-consumption—blood alcohol concentration (BAC). Our findings indicate that cannabis impairs psychomotor and cognitive functions and that THC serum concentrations are roughly correlated with driving impairment. However, we were unable to define a clear cut-off-value above which a significant increase in impairment could be detected. Slightly (but statistically significant) higher THC serum concentrations and CIF values were observed in cases where there was concrete evidence of criminal offenses pursuant to § 316 of the German Penal Code. However, absolute differences were small and there was too much overlap between the two groups to define a useful cut-off-value for THC. There is a measurable relationship between THC serum concentration and impairment. This relationship is too weak to scientifically define a cut-off value. Regarding the revised legal threshold for THC serum concentration, we found that approximately one-third of the evaluated cases would no longer be subject to legal penalties if the new threshold of 3.5 ng/ml were applied.

## Introduction

Cannabis and alcohol are the main substances detected among road users [1, 2] and the prevalence of driving under the influence of cannabis (DUIC) is on the rise [3]. According to the European Drug Report 2025 published by the European Monitoring Centre for Drugs and Drug Addiction [4] an estimated 24 million individuals in the European Union reported cannabis use in the previous year, with 4,3 million consuming it daily or almost daily [4].

While the effects of specific blood alcohol concentrations on driving performance are widely studied, the impact of defined THC concentrations remains controversial, and legally defensible threshold values for cannabis use are difficult to determine [5, 6]. In general, cannabis negatively affects reaction time, concentration and psychomotor functions [3, 7]. DUIC has been associated with increased crash risk [8], impaired cognition, and adverse effects on mental health [9]. Moreover, it can lead to difficulties in lane keeping and fluctuations in driving speed, especially within the first hour following consumption [1, 10]. Although cannabis-induced psychomotor and driving impairments appear to be dose-dependent, the dose-impairment relationship is highly complex [7, 11]. While Albrecht et al. [12] showed an increased crash culpability risk at THC blood concentrations from up to approximately 1.5 ng/ml – 3 ng/ml with a doubling risk at 5 ng/ml (and a quadrupling risk at 10 ng/ml), White & Burns [13] were not able to detect credible evidence of an increased crash risk associated with prior THC consumption by conducting systematic research.

According to Brubacher et al. [3], whole blood concentrations of delta-9-tetrahydrocannabinol (THC), exceeding 5 ng/ml are associated with a higher crash risk. Similarly, Hartman et al. [14] reported that THC blood concentrations between 2 and 5 ng/ml correlate with early signs of driving impairment, and Ramaekers et al. [15] also found observable increases in impairment within a similar THC serum concentration range.

The co-consumption of cannabis and alcohol further amplifies effects on driving ability [7, 16] and is linked to an increased risk of fatal crashes [17, 18]. Although the literature remains divided on whether these effects are additive or synergistic [19], substantial evidence suggests that driving under the influence of alcohol and cannabis (DUIAC) can result in impaired lane control, delayed reaction times, and erratic driving behaviour [1]. On the other hand, however, a systematic literature search by White & Burns 2023 [20] could not provide convincing proof for an exacerbation of the alcohol-related effects on crash risk due to a co-consumption of cannabis.

In light of worldwide tendencies towards cannabis legalization, there is a growing need for a better understanding of the impairing effects of cannabis – alone or in combination with alcohol – on driving ability [1]. In Germany the annual prevalence of DUIC among individuals aged 15 to 34 years was estimated to be 17.2% in 2021 [21].

Until recently, German law classified a THC serum concentration of at least 1 ng/ml an administrative offense according to § 24a of the German Road Traffic Act. The same statute applies to drivers with a breath alcohol concentration (BrAC) of at least 0.25 mg/l or a blood alcohol concentration (BAC) of at least 0.50 g/kg (while driving a motor vehicle). A BAC of at least 0.30 g/kg may qualify as a criminal offense if alcohol-related impairments are observed. A criminal offense under § 316 or § 315c of the German Criminal Code is defined by a BAC of at least 1.10 g/kg, even without any signs of impairment (“per se” law). Unlike for alcohol, there are no defined absolute or relative legal thresholds for other psychoactive substances in the context of criminal liability.

Cannabis has been partially legalized in Germany in April 2024. A THC-level equivalent to 0.5 g/kg BAC could not be established due to its complex pharmacokinetics [22]. Instead, a new threshold based on the findings of Ramaekers et al. [15] and Irwin et al. [23] was established, assuming that THC serum concentrations between 2 and 5 ng/ml may go along with signs of psychomotor impairment comparable to those observed at a moderate BAC (e.g. 0.2 g/kg). After deliberation, a value of 3.5 ng/ml was proposed and subsequently adopted as the new legal threshold limit [24].

In this context we investigated our hypotheses that.

- driving impairments are predominantly found in drivers with high THC serum concentrations (> 10 ng/ml).
- the medical examination of suspected delinquents solely consuming cannabis does not allow for conclusions about THC serum concentrations.
- the co-consumption of cannabis and alcohol can lead to unpredictable, serious impairments even at rather low concentrations.
- apart from considering THC serum concentrations, the cannabis influence factor (CIF) may help to differentiate between impaired and unimpaired drivers.

## Materials and methods

Cases involving general traffic controls, traffic offenses or accidents collected between 01/01/2020 and 12/31/2021 were included in this study. In Germany, during such incidents, an initial on-site assessment is conducted by police officers, who document observable indicators for impairment including driving behaviour, demeanour, gait, facial expression, speech, physical anomalies, level of consciousness, pupillary response, time perception and results from coordination tests (finger-to-finger and finger-to-nose test). If the initial tests are indicative of a DUI-offence and blood sampling becomes necessary, a second, more detailed medical examination is conducted by police-appointed physicians, covering similar categories and including additional assessments.

### Inclusion and exclusion criteria

Only cases in which cannabis alone or in combination with alcohol was detected in our toxicological screening were included (*n* = 796). Toxicological analysis hereby consisted of an immunochemical screening for amphetamines (amphetamine, methamphetamine and designer amphetamines), cannabinoids, cocaine (and metabolites) and opioids, as well as a highly specific, chromatographic confirmatory analysis for any positive findings.

Cases involving cyclists (*n* = 7) and cases with clearly inconsistent and anomalous assessments by a single physician (*n* = 45) were excluded. Additionally, four cases were excluded due to missing incident data. The final dataset comprised 740 cases: 695 involving driving under the influence of cannabis (DUIC) and 45 involving combined cannabis and alcohol use (DUIAC).

### Assessment of impairments

Behavioural and driving impairments were categorized based on documentation from police and medical evaluations. Behavioural impairments were classified into eight categories:

- mood (e.g. euphoric, indifferent),
- behaviour (e.g. aggressive, disinhibited),
- consciousness (e.g. apathetic, disorientated),
- general motor skills (e.g. slowed down),
- gait/posture (e.g. staggering, tendency to fall),
- speech (e.g. slurred, slowed down),
- cognition (e.g. problems of concentration or reaction),
- and others.

Driving impairments were classified into eleven categories:

- lane-keeping difficulties (e.g. swerving),
- erratic driving style (e.g. unsafe driving, loss of control),
- improper overtaking (e.g. risky overtaking manoeuvres),
- speed (e.g. exceeding the speed limit, reduced speed),
- traffic light violations (e.g. red-light violation),
- lighting violations (e.g. driving without light in the dark),
- other traffic rule violations,
- accidents,
- failure to maintain distance,
- turning errors,
- and other behaviours.

“Hard” criteria were defined – in concordance with German supreme court jurisprudence – both subjectively (e.g., “confusion,” “drowsiness,” “disorientation,” “slurred speech”) and objectively (e.g., involvement in a collision).

Furthermore, data were analysed for age, sex, day and time of incident, choice of motor vehicle (car, motorbike, e-scooters) and correlated with the measured serum concentrations of THC and metabolites (THC-COOH, THC-11-OH) and/or BAC in blood serum. Where possible, CIF values were calculated and the degree of habituation for cannabis use was assessed (with reference to Daldrup et al. [25] >150 ng/ml THC-COOH indicating regular users).

The CIF value reflects the ratio of active THC to its metabolites (THC-OH and THC-COOH) [26]. In accordance with Daldrup et al. [26] CIF values below 10 were interpreted as indicative of the subacute elimination phase, those exceeding 10 as a sign for recent cannabis use. CIF values were rounded to whole numbers, with a maximum value of 30, as recommended by Daldrup [27].

Cases were grouped based on (preliminary) legal classification: those classified as putative administrative offense under § 24a of the German Road Traffic Act (“§ 24a-group”) and those involving specific evidence of a criminal offense under § 316 or § 315c of the German Criminal Code (“§ 316-group”). Among the 740 cases, 546 were classified as putative administrative offenses (§ 24a), and 194 represented potential criminal offenses (§ 316).

### Statistics

First, the frequency of each behavioural and driving impairment category was evaluated and Fisher’s test was used to calculate p-values. Odds ratios (ORs) were then determined for each category.

To examine whether THC serum concentration or CIF values were significantly associated with impairments, logistic regression models and Wald tests were employed. Differences in THC serum concentrations and CIF values between the § 24a and § 316 groups were assessed using t-tests.

For DUIAC cases, the effect of alcohol on driving and behavioural impairments was further analysed using logistic regression with Wald tests. The interaction between BAC and THC serum concentration (and between BAC and CIF) was assessed using the following logistic regression models:

$$\begin{array}{l}\mathrm{logit}\left(P\left(\mathrm{impairment}\right)\right)\\={{\upbeta\:}}_{0}+{{\upbeta\:}}_{1}\times\:\text{THC serum concentration}+{{\upbeta\:}}_{2}\times\:\mathrm{BAC}+{{\upbeta\:}}_{3}\\\times\:\text{THC serum concentration}\times\:\mathrm{BAC}\end{array}$$

$$\:\mathrm{logit}\left(P\left(\mathrm{impairment}\right)\right)={{\upbeta\:}}_{0}+{{\upbeta\:}}_{1}\times\:\mathrm{CIF}+{{\upbeta\:}}_{2}\times\:\mathrm{BAC}+{{\upbeta\:}}_{3}\times\:\mathrm{CIF}\times\:\mathrm{BAC}$$

## Results

707 of the 740 cases involved male subjects, only 33 involved female subjects. The overall median age was 28.6 years with a range from 15 to 62 years. A majority of the cases (*n* = 569) involved individuals under the age of 34. No significant variation in the frequency of incidents was observed across different weekdays.

In most cases (*n* = 730) the type of vehicle was specified (or plausibly assumed to be) a car. 10 cases concerned motorbikes (*N* = 4) or e-scooters (*N* = 6).

In view of the newly established THC legal threshold in Germany (3.5 ng/mL, replacing the previous 1 ng/mL limit), we examined its potential impact on legal classifications. 182 of the 546 cases (33,3%) classified as suspected violations of § 24a (excluding the cases with a co-consumption of alcohol) showed a THC serum concentration under 3.5 ng/ml (with one of them having a THC serum concentration under 1 ng/ml).

### Age, THC serum concentration, CIF value

For a better understanding of the effects of cannabis on driving, we first analysed the 695 DUIC cases’ distribution of age, THC serum concentration and CIF value, excluding cases in which cannabis in combination with alcohol was found (*n* = 45).

**Fig. 1:**
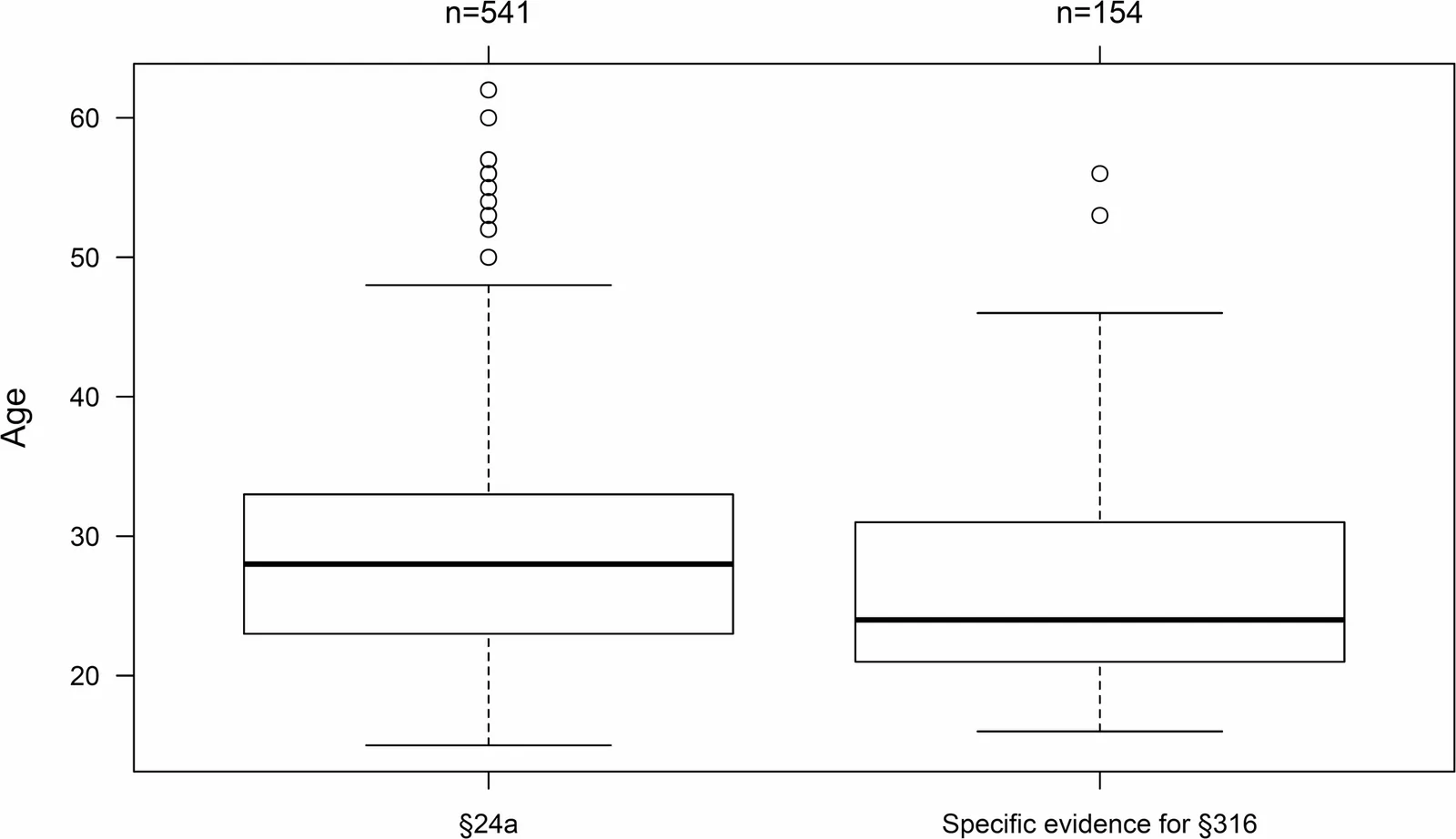
Boxplot for age distribution of exclusively THC positive cases (cases with co-consumption of alcohol excluded); 541 cases being medicolegally classified as administrative offense (§ 24a) and 154 cases as potential criminal offenses (§ 316). The black line indicates the median, the box includes 50% of all values. The circles indicate outliers

As illustrated in Fig. 1, the age distribution was similar between the administrative offense group (§ 24a) and the criminal offense group (§ 316), although slight differences were observed. The overall median age in the dataset was 27 years, with a mean age of approximately 28.5 years. The § 24a group had a median age of 28 years (interquartile range (IQR): 23–33 years), whereas the § 316 group showed a median of 24 years (IQR: 21–31 years).

**Fig. 2:**
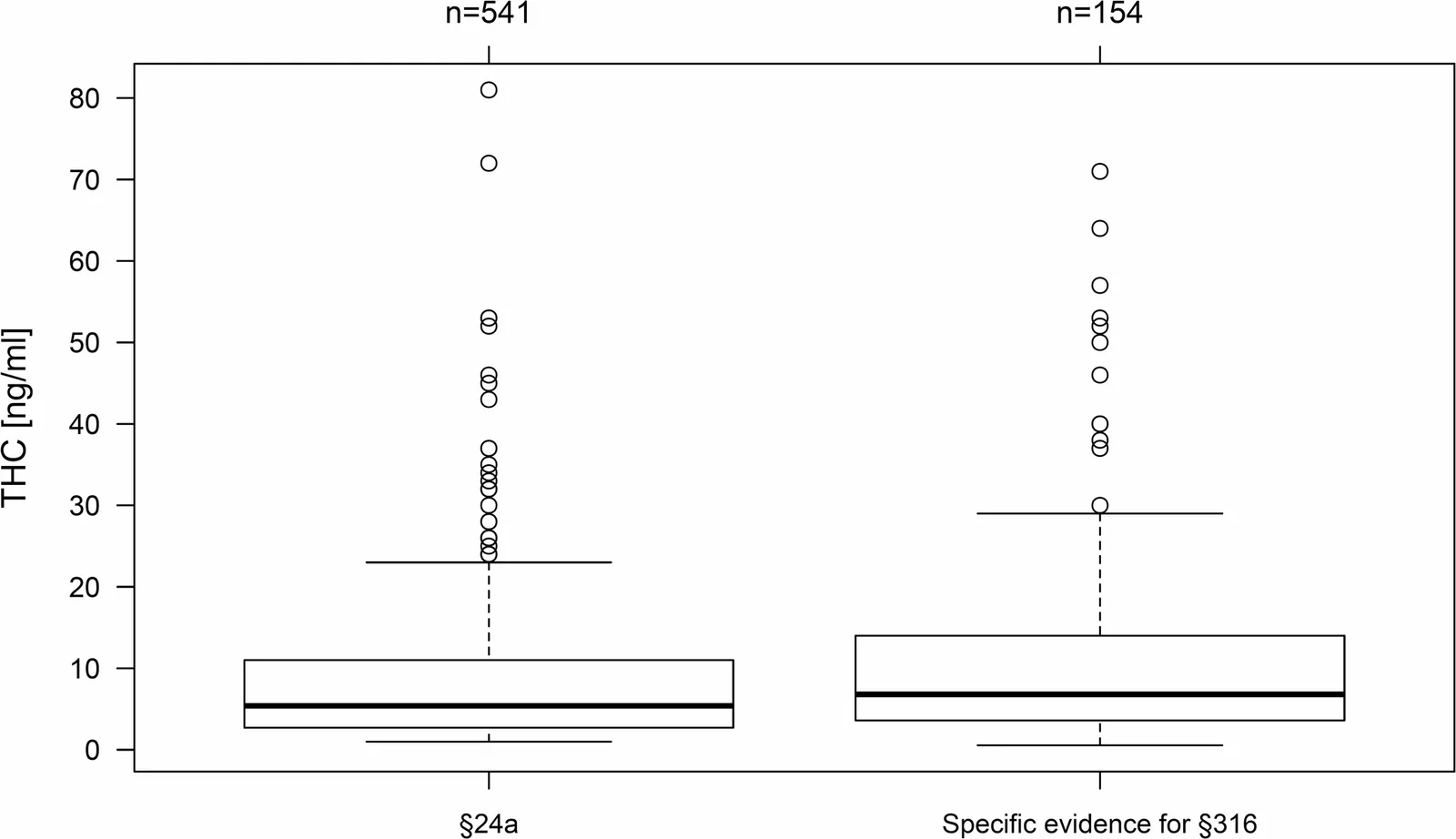
Comparison of the distribution of the THC serum concentration between the groups medicolegally classified as administrative offenses (§ 24a) and potential criminal offenses (§ 316)

**Fig. 3:**
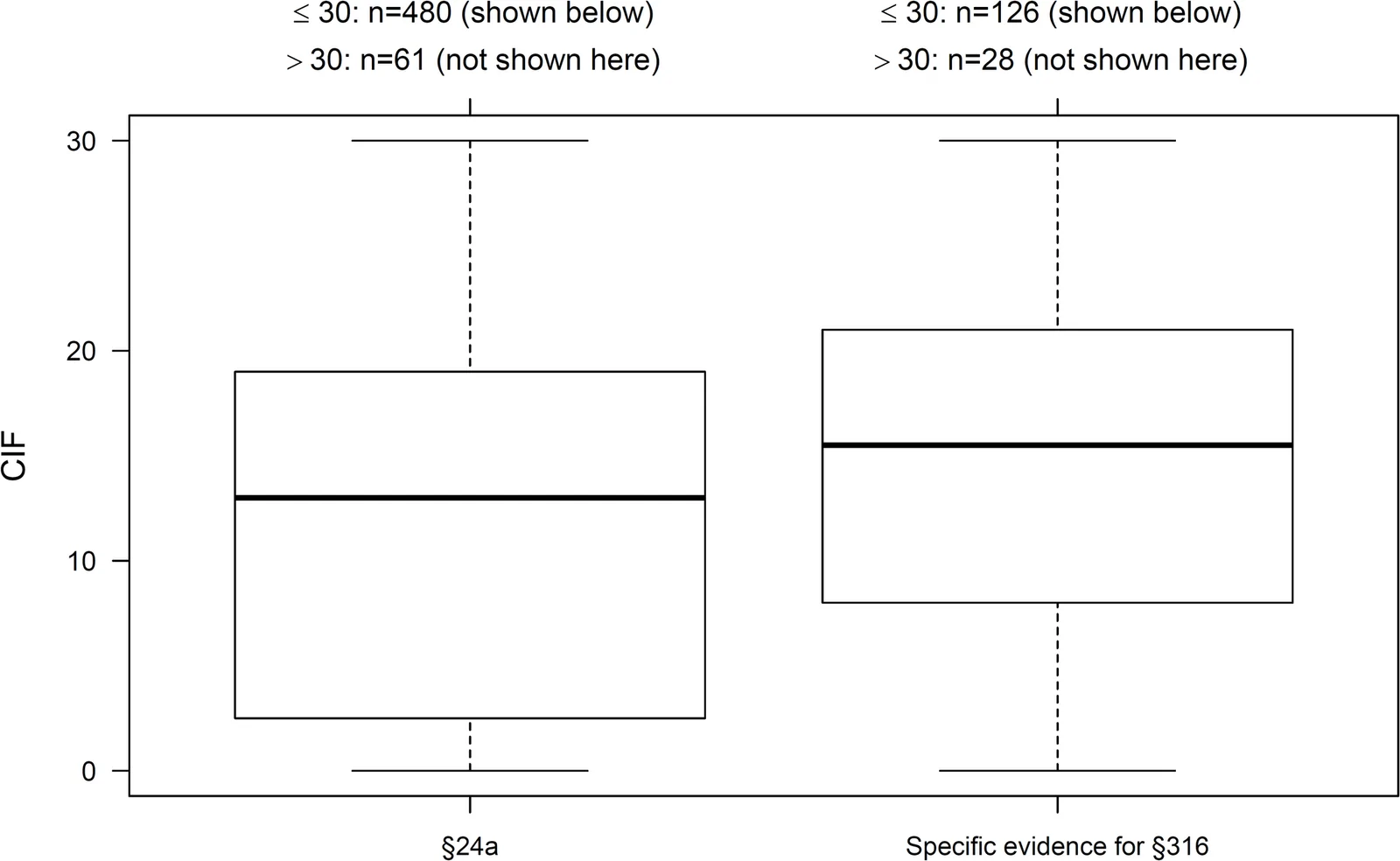
Comparison of CIF value distribution in both groups. The case number differs slightly, as in some cases not all metabolites were measurable and CIF, therefore, not calculable

The group with an exclusive influence of cannabis with specific evidence for criminal offenses (§ 316) from a medicolegal point of view showed higher mean THC serum concentrations and CIF values as illustrated in Figs. 2 and 3:

The median THC serum concentration in the § 24a group was 5.4 ng/ml (interquartile range [IQR]: 2.7–11 ng/ml), with a mean value of approximately 8.39 ng/ml. The § 316 group exhibited a higher median concentration of 6.8 ng/ml (IQR: approximately 3.63–14 ng/ml), and a higher mean value of approximately 11.53 ng/ml.

To assess the distribution of the Cannabis Influence Factor (CIF), values were capped at a maximum of 30, in line with established methodological recommendations. The median CIF value in the § 24a group was approximately 13 (IQR: 3–19; mean value: 12), while the § 316 group showed a higher median CIF of approximately 15.5 (IQR: 8–21; mean value: 14). Notably, in 61 cases (11.3%) from the § 24a group and 28 cases (18.2%) from the § 316 group, the calculated CIF exceeded the upper threshold of 30.

**Fig. 4:**
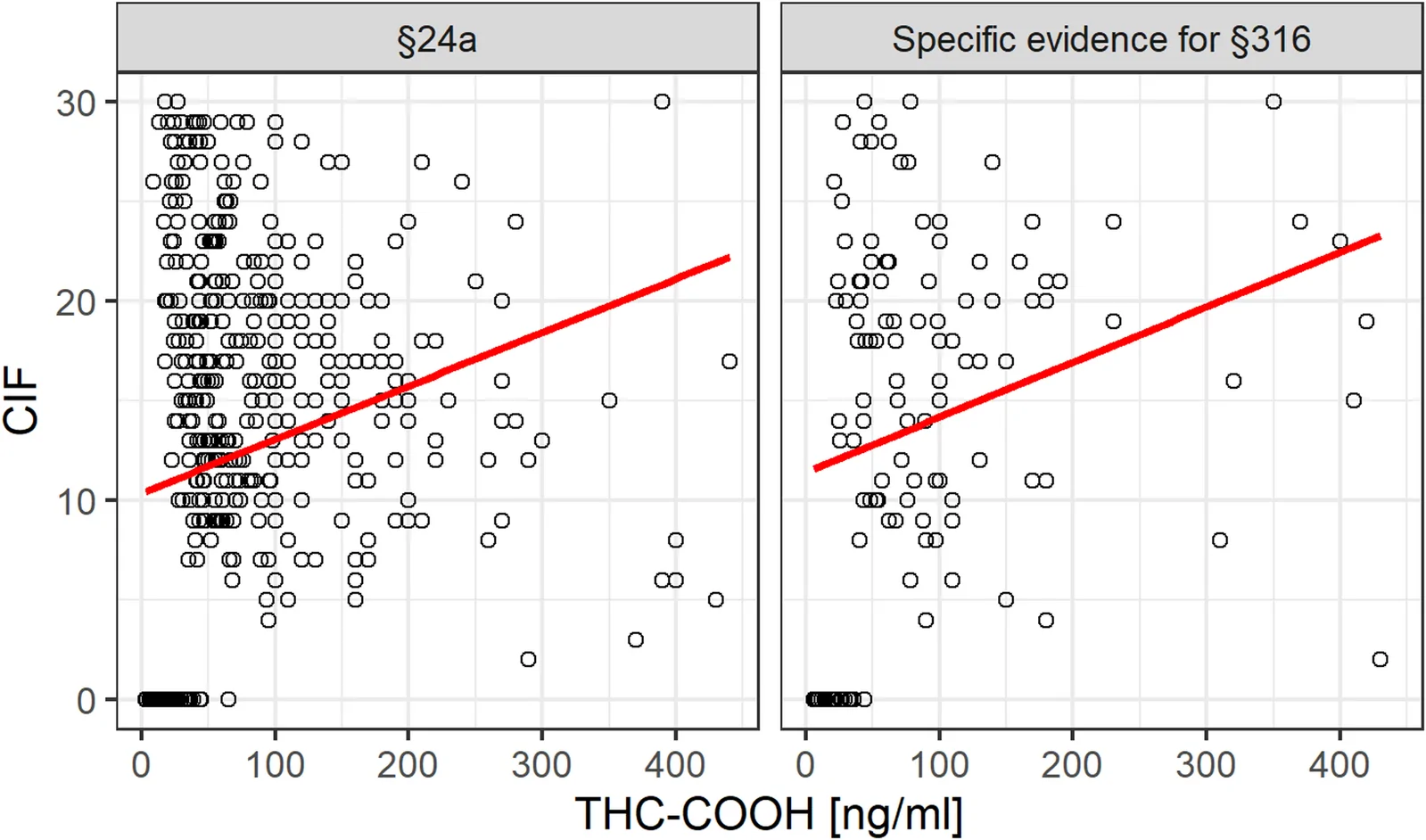
Comparison of the coherent CIF and THC-COOH (ng/ml) values of each case in both groups with a line of best fit

Given the higher mean/median CIF values observed in cases with specific evidence of criminal offenses (§ 316 group), we further examined THC-COOH serum concentrations in both groups to explore a potential link to regular cannabis use and its contribution to the CIF. As shown in Fig. 4, no clearly differential pattern (e.g. clusters of high-CIF/low-THC-COOH in the § 316-group or low-CIF/high-THC-COOH in the § 24a-group) can be observed between the two groups.

### Impairments

In order to identify driving and behavioural impairments that are solely due to cannabis consumption, we excluded cases with an additional consumption of alcohol (analysed *n* = 695 cases).

A total of 125 cases with driving impairments and 219 cases (double mentioning possible) with behavioural impairments were found.

Regarding the driving impairments, 70 of the 125 (56%) cases were presenting “hard criteria” for a criminal offense (e.g. loss of vehicle control, swerving, lane departure or use of multiple lanes, red light violations, single-vehicle accidents, collisions with vehicles, rear-end collisions, violations of right-of-ways) with most of them being abnormal speed, followed by accidents and lane-keeping violations.

Of the 219 behavioural impairments, 107 (49%) were classified as “hard criteria” for a criminal offense, most of which were found in the cognition, consciousness, mood and gait/posture categories.

Despite these findings, no statistically significant relationship could be established between either THC serum concentration or CIF value and the presence of driving impairments. The same was true for behavioural impairments, despite a significant difference between the two groups in the category “consciousness” with significantly more subjects described as “confused” in the § 316 group.

### Relationship between § 316 (criminal offenses) and THC serum concentration – distribution

To further explore the relationship between THC serum concentration and impairments according to a criminal classification under § 316, we analysed all 194 cases with specific evidence of criminal offenses—including those involving co-consumption of alcohol.

**Fig. 5:**
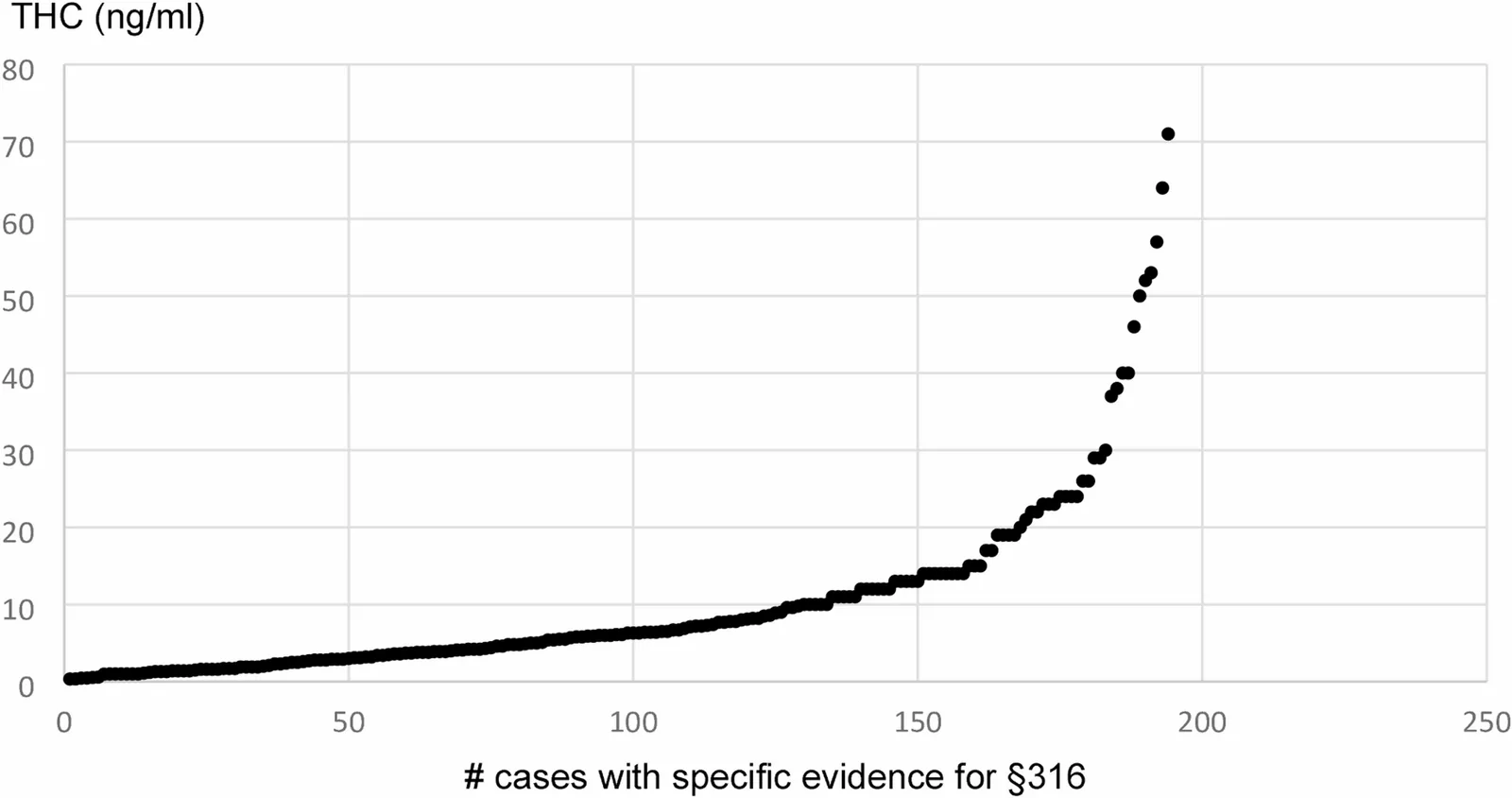
All cases (including co-consumption of alcohol) with specific evidence for criminal offenses (§ 316) in correlation to the measured THC serum concentration (*n* = 194)

As shown in Fig. 5, the majority of these cases (*n* = 134) had THC serum concentrations below 10 ng/mL, although outliers reached values exceeding 70 ng/ml. The mean THC serum concentration in this group was approximately 10 ng/mL, while the median value was 6.1 ng/ml.

Using t-tests, cases with specific evidence of criminal offenses exhibited significantly higher THC serum concentrations compared to cases classified as administrative offenses (*p* < 0.01). A similar, statistically significant difference was observed for CIF values (*p* = 0.045).

### Co-consumption with alcohol

A total of 45 cases showed positive blood alcohol and THC serum concentrations. BACs varied from 0.4 g/kg to 2.41 g/kg with a median of 1.31 g/kg. Six cases were classified as administrative offenses, whereas 39 cases showed specific evidence for criminal offenses.

The cases involving co-consumption presented a total of 162 driving impairments (100 impairments or 62% of those being classified as “hard criteria”) with most of them in the categories “speeding violation”, “lane keeping violation” and “accident”, showing a significant correlation for lane keeping violations and accidents.

**Fig. 6:**
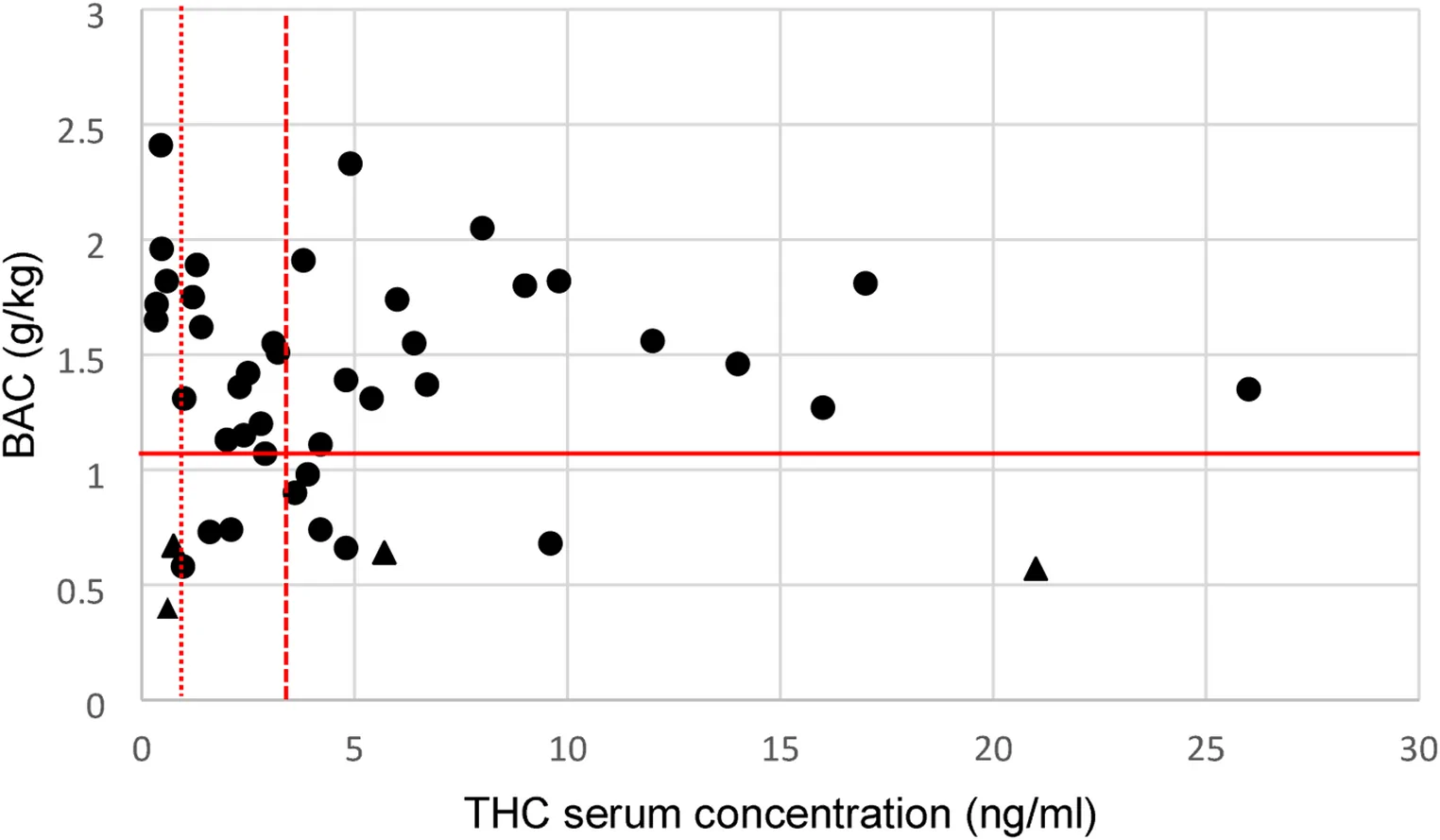
Cases with co-consumption of cannabis and alcohol in correlation with the measured blood alcohol concentration (BAC) and THC serum concentration (*n* = 45). Cases classified as putative administrative offenses were illustrated as triangles (*n* = 4), whereas cases with specific medicolegal evidence for criminal offenses were illustrated as dots (*n* = 41). The horizontal red line marks a BAC of 1.1 g/kg, the vertical red lines mark THC serum concentration of 1 ng/ml resp. 3.5 ng/ml

As illustrated in Fig. 6, the majority of co-consumption cases showed specific medicolegal evidence for criminal offenses (41 out of 45 cases). In most cases, BAC alone already explains the criminal offense (“per se”-law). Of the four cases classified as putative administrative offenses, most showed no documented driving or behavioural impairments. In the one case where impairments were noted, further toxicological analyses were recommended. These four cases had BACs ranging from 0.4 g/kg to 0.67 g/kg. In contrast, all cases within the § 316 group demonstrated at least one documented driving and/or behavioural impairment. However, no statistically significant interaction was observed between THC serum concentration and BAC with regard to the likelihood or severity of impairments.

Only one case — presenting no compelling signs for a criminal offense — had a BAC below 0.5 g/kg with a THC serum concentration of 0.61 ng/ml. All other cases with co-consumption had a BAC above 0.5 g/kg, with 32 cases having a BAC above 1.1 g/kg. The THC serum concentration ranged between 0.34 ng/ml and 26 ng/ml with only 8 cases showing a THC serum concentration below 1 ng/ml (and 22 cases showing a THC blood concentration of less than 3.5 ng/ml).

## Discussion

Our analyses aimed to identify the respective thresholds for the THC serum concentration and CIF, above which impairment levels significantly increase.

We analysed a dataset comprising 740 cases of suspected impaired driving, with a strong predominance of young male individuals (707 males, 569 subjects under 34 years, median age: 28.6 years). These demographic trends support the findings of Capler et al. [7] and Brubacher et al. [3], who also identified young adult males as being at increased risk for driving under the influence of cannabis (DUIC) and for exhibiting higher THC serum concentrations.

Consistent with other studies (e.g [1, 28, 29])., our results support the hypothesis that cannabis affects psychomotor functions and cognition, and is correlated to driving impairment as evidenced by speeding and lane keeping violations as well as crashes. Various studies have shown a high risk for (fatal) crashes and a contribution of cannabis/DUIC to crashes (e.g [8, 30, 31]). Similar to the effects of cannabis on lane keeping, studies using driving simulators showed an increase of SDLP (standard deviation of lateral position) after consumption of cannabis, indicating a positive correlation between increasing THC serum concentrations and increasing SDLP [2, 7, 10, 19].

The growing evidence of more frequent drug-induced performance impairments among younger drivers may be due to their limited ability to compensate through driving experience, and supports a low THC limit of e.g. 1 ng/ml for novice drivers as currently implemented in German law.

In this context, the complex pharmacokinetics of cannabis have to be considered: as Pearlson et al. [5] stated, consumption mode (inhalation versus oral ingestion), dosage and frequency of consumption are influencing factors for both THC-concentrations and, more importantly, THC-effects. Due to its lipophilic nature, THC is rapidly distributed to highly perfused organs such as the brain, heart and liver [5]. It is then stored in fatty tissue as well as organs with a high amount of fat, where it accumulates with frequent consumption and is then gradually released over a longer period of time [5]. Therefore, the blood serum concentration of THC at a given time does not necessarily reflect the dosage consumed, nor is it strictly correlated with the level of impairment [5]. Thus, a linear concentration-impairment-relation does not exist for cannabis [5, 10, 32]. On the contrary, the degree of impairment seems to underlie various factors, one of the most important being the time between most recent cannabis consumption and driving (Tank et al. [32]). In this context, it is important to note that the detection of low levels of THC and its metabolites in blood samples does not prove an acute cannabis influence [5]: due to accumulation, regular/chronic consumers might present with THC serum concentrations above 1 ng/ml even after longer periods of abstinence in the sense of so-called “residual THC” [33, 34].

Our hypothesis that CIF could distinguish between impaired and unimpaired drivers was not supported, as there was no clearly discernible difference in CIF-patterns between the two groups: in contrast to our initial expectations, no characteristic clustering of high-CIF/low-THC-COOH in the § 316-group or low-CIF/high-THC-COOH in the § 24a-group was observed. Conversely, neither the incident nor the psychophysical examination of suspected delinquents/DUIC are reliably related to the THC serum concentration: Our analysis revealed no significant correlation between THC-concentration and/or CIF values and the presence of specific behavioural or driving impairments.

The subgroup of co-consumption cases (*n* = 45) showed multiple behavioural and driving impairments. However, most probably due to the small sample size, no statistically significant interaction between THC serum concentration, BAC, and the degree of impairment could be established. Despite this, our findings align with those of Downey et al. [16], Fares et al. [1] and Dubois et al. [18], who noted that combined use of alcohol and cannabis has additive or synergistic effects, increasing the risk of crashes and severe impairments. 62% of the driving impairments found in our co-consumption cases were classified as “hard criteria” and a significant correlation for lane keeping violations and accidents could be shown. As an outlook, The Effects of Cannabis Legalization (ECaLe) Study [35] suggests that there might be an increase in the number of THC-positive drivers after legalization, as well as an increased prevalence of fatal crashes involving drivers with positive cannabis blood test results. Similar findings could be observed in some states of the USA and Canada after legalization: according to González-Sala et al. [36], the prevalence of DUIC in Washington increased from 22.2% to 32.7% after legalization, accompanied by an increase in fatal crashes involving cannabis. However, literature on the subject remains inconclusive, as some studies found no significant changes in traffic outcomes post-legalization. Thus, further research will be necessary to clarify the effects of German cannabis legalization.

### Limitations

Our dataset comprised 740 cases with confirmed positive THC serum concentrations, including 45 cases involving co-consumption of alcohol. All cases were generated during traffic controls by the police. Given the relatively small number of co-consumption cases, this subgroup may not be representative of the full spectrum of possible impairments related to simultaneous cannabis and alcohol use.

The evaluation of impairment relied primarily on police reports and assessments conducted by contract physicians. In cases where the quality or consistency of the assessment was questionable, such findings were excluded from analysis. However, the time interval between the incident and blood sample collection was not standardized in this routine setting, which may have influenced measured THC serum concentrations due to the rapid pharmacokinetics of the substance.

In some instances, not all case documentation was available, and therefore, revisions of the preliminary assessments may be warranted if additional information became accessible.

Furthermore, analyses for other centrally acting substances (including new psychoactive substances) were only conducted in selected cases where observed impairment could not be explained by cannabis and/or alcohol alone and (additionally) only if explicitly commissioned by the police; such analyses were not performed routinely – not least because they were not deemed sensible in all cases from an expert’s point of view.

When assessing § 316 cases with co-consumption, it must be considered that a criminal offense was often committed based on BACs alone, given that the BACs were at least 1.1 g/kg in 32 cases.

Finally, no control group—such as drivers under the influence of alcohol alone—was included for comparative purposes. Future studies incorporating such controls would strengthen causal interpretation and allow for a clearer delineation of the specific effects of cannabis, alcohol, and their combination on driving performance.

## Conclusions

This study was unable to establish a cut-off concentration for THC in serum, above which driving impairment could be reliably detected. Furthermore, no significant correlation was observed between THC serum concentrations or CIF values and documented impairments.

The failure to define a cut-off was due to the fact that both THC serum concentrations and CIF values were significantly, yet minimally, higher in cases involving specific evidence of criminal offenses. While this suggests a potential association with more severe impairment or higher levels of substance influence, a notable overlap between the two groups makes it impossible to define a reliable cut-off that would exclude every single fit driver.

Given the complex pharmacokinetics of cannabis, variability in individual response, and inconclusive evidence in the current literature, we recommend further research to better understand the effects of cannabis on driving performance. In particular, long-term and controlled studies are needed to address traffic safety questions.

## Key points

1. Retrospective analysis of cases with suspected DUICs or DUIACs.
2. Evaluation of the relation between THC serum concentration and signs of impairment.
3. Evaluation of CIF as a potential marker to differentiate between impaired and unimpaired drivers.

## Data Availability

The datasets generated during and/or analysed during the current study are available from the corresponding author on reasonable request.
